# Supplementary material for: “Sickness has no time”: Awareness and perceptions of health care workers on universal health coverage in Uganda
Source: PLoS One. 2024 Jul 18;19(7):e0306922. doi: 10.1371/journal.pone.0306922 (PMC11257248; doi:10.1371/journal.pone.0306922)
Supplement: S1 Appendix — (PDF) [file pone.0306922.s001.pdf]

S1 Appendix. Online UHC survey – background file for Qualtrics^{XM}

Data collection tool – Online survey for all

Background information concerning online survey
This survey will address more generalised topics and potentially sensitive questions, as it is anonymised. The format includes Likert scale questions, drop down selections and open-ended questions, some of which will be optional to remain within a 15-minute timeframe. The responses from this survey will also aim to refine the final semi-structured interviews.

Introductory information displayed to the respondent

Welcome
Thank you for choosing to participate in this survey on universal health coverage (UHC). This survey is conducted by Susan Ifeagwu, as part of a PhD research project at the Department of Public Health and Primary Care at the University of Cambridge.

This survey aims to explore the perceptions, understanding and priorities of UHC in low- and middle-income countries (LMICs), in general terms as well as in context of Uganda. However, expertise in Uganda is not a prerequisite for participation in this survey. This survey will address generalised topics related to UHC and will remain anonymised. The format includes Likert scale questions, drop down selections and open-ended questions, some of which are optional.

The survey is anonymised and should take approximately 10 to 15 minutes to complete. Kindly note that all questions do not need to be answered and it is possible to move on with the rest of the survey should this be the case. Please do not hesitate to contact the researcher (sci24@medschl.cam.ac.uk or sci24@cam.ac.uk) if you have any comments or queries.

Before you begin
To proceed to the survey, please click on the next page where you will find further information and an informed consent statement. Please also click on the upcoming CAPTCHA verification and follow the instructions.

Thank you very much for your time and consideration.

Survey Title
Perceptions and values related to universal health coverage (UHC)

Why you have been invited to participate
You have been invited to participate in this research project on the basis of your work and expertise in the area of UHC, health policy, financing, and/or health systems research.

Type of data collected
The online survey will provide us with your completely anonymous answers to questions about UHC in Uganda. At the end of the questionnaire you will be asked whether you could participate further by taking part in more detailed interviews or focus group discussions. This is entirely up to you but, if you do, we will need you to submit your contact details. These will be stored securely and not linked with your answers on the online survey or interview/focus group responses. If you take part in interviews/focus groups, these will be recorded on a hand-held digital recorder, so that we have an accurate record of what was discussed. We will then type up the transcript, making sure that any personally identifiable information is not included. All interviews will be conducted in English.

European Union General Data Protection Regulation (EU GDPR) Compliance
Data security during and after the study will comply with the EU GDPR. The researcher has completed the online GDPR training, made available by the Personal and Professional Development Training Programme of the University of Cambridge. Qualitative data collected will be anonymised in the form of coding to remove any personal identifiers and ensure confidentiality. Moreover, data will be saved and stored in an anonymised way both during and after the completion of the study, through the University of Cambridge's secured OneDrive system and the departmental computer storage. All data collected abroad will also be encrypted and stored in a password protected external USB hard drive. Data will then be transferred safely to Cambridge Public Health at the University of Cambridge, where it will be stored in a locked filing cabinet with access limited to the researcher and co-principal investigators.

Following completion of the study, the anonymised data will also be shared through the University of Cambridge data repository platform. The anonymised research data, consent forms and transcript records will be retained up to 10 years after the publication of the data, as required for the Cambridge repository platform, Apollo. Data will be stored in the form of coding, a participant number, and no other personal identifying information will be included. General information about how the University uses personal data can be found here: https://www.information-compliance.admin.cam.ac.uk/data-protection/applicant-data.

Publication of data
Anonymised extracts may be used in the final written report and any potential publications.

Withdrawing from the research study
You have the opportunity to terminate participation and withdraw from the study at any time with no adverse consequences. In order to withdraw from the study, you can send an email to the researcher (sci24@cam.ac.uk). The time limit for withdrawal would be up to 6 months following participation. You also have the right to decline information offered at any point.

Details of possible risks associated with participation
There are no known risks associated with participating in this research study. Strict confidentiality and anonymity will be maintained throughout the course of the research project and final reporting to avoid any possible risks. However, should any issues arise, you have the possibility of contacting the University of Cambridge.

Ethical review of the study
This project has been reviewed by the University of Cambridge Psychology Research Ethics Committee, the Scientific Review Committee at the Infectious Diseases Institute, the Makerere University School of Public Health Research Ethics Committee Institutional Review Board and the Ugandan National Council for Science and Technology.

Thank you for considering participating in this research study.

Informed Consent
I hereby declare that I have read and understood the abovementioned information. I agree with the terms of participation for this survey and consent to the analysis of my responses.

- Yes
- No

Captcha verification

- I'm not a robot (AUTO GENERATED IMAGE APPEARS)

Questions

General introductory question

1. What is your profession?
   i. Health care worker
   ii. Policymaker
   iii. Other (please specify)

**Note:* based on the response to the above, the questionnaire will be divided to either follow Strand A, the more generalised stakeholder survey at the international, regional and national level, or Strand B, the health care worker survey at the local level. The answers (ii) and (iii) will lead to Strand A, while (i) will lead to Strand B.

The following questions for Strand A and B are provided below.

Strand A: stakeholder survey for the international, regional and national levels

2. Please select the type of institution or organisation you work with.
   a. Academia
   b. Civil Society
   c. Government
   d. Non-Governmental Organisation (NGO)
   e. Private/Industry
   f. United Nations (UN) Organisation
   g. Other (please specify)

3. Which areas do you work in?
   a. Communicable Diseases
   b. Essential Medicines
   c. Emergencies and Humanitarian Settings
   d. Development
   e. Health Systems
   f. Human Rights
   g. Financing
   h. Non- Communicable Diseases
   i. Other (Please specify)

4. At what level does your institution or organisation operate?
   a. International
   b. Regional
   c. National
   d. District
   e. Other (please specify)

5. Which country does your institution or organisation work in?
   a. *Drop down options for countries*.

Specific

6. Which of the following definitions would you say is nearest to UHC?
   a. All people have access to health care services they need.
   b. All people have access to health care services they need, of sufficient quality to be effective.
   c. Some people have access to health care services they need, without facing financial hardship.

d. All people have access to health care services they need, of sufficient quality to be effective and without facing financial hardship.
e. All people have free coverage.
f. Other (please specify)

7. What level of importance would you value UHC? *[Likert scale]*
    a. Very important
    b. Quite important
    c. Fairly important
    d. Not important
    e. N/A

8. How do you think policymakers at country level value UHC? *[Likert scale]*
    a. Very important
    b. Quite important
    c. Fairly important
    d. Not important
    e. N/A

9. How do you think policymakers at international level value UHC? *[Likert scale]*
    a. Very important
    b. Quite important
    c. Fairly important
    d. Not important
    e. N/A

10. What is the reasoning for your selection (*optional*)?
    [open answer format]

11. What would you say are some of the barriers to achieving UHC in Uganda?
    a. Infrastructure
    b. Human resources
    c. Financing
    d. Awareness
    e. Collaboration
    f. Political economy
    g. Information
    h. Other reasons (please specify)

12. Which country would you describe as best practise in terms of their approach towards achieving UHC?
    [open answer format]

13. What do you consider most important in order for Uganda to achieve UHC? *(multiple selections possible)*
    a. Awareness
    b. Collaboration
    c. Expertise
    d. Financing
    e. Human resources
    f. Information
    g. Infrastructure
    h. Political will
    i. Other reasons (please specify)

14. Do you think that UHC would have an impact on…? *[Likert scale]*

The 17 Sustainable Development Goals (SDGs)	Worsen (1)	No effect (2)	Not sure (3)	Likely/may have an impact (4)	Extremely likely/high impact (5)
SDG 1: No Poverty<br>SDG 2: Zero Hunger<br>SDG 3: Good Health and Well-being<br>SDG 4: Quality Education<br>SDG 5: Gender Equality<br>SDG 6: Clean Water and Sanitation<br>SDG 7: Affordable and Clean Energy<br>SDG 8: Decent Work and Economic Growth<br>SDG 9: Industry, Innovation and Infrastructure<br>SDG 10: Reduced Inequality<br>SDG 11: Sustainable Cities and Communities<br>SDG 12: Responsible Consumption and Production<br>SDG 13: Climate Action<br>SDG 14: Life Below Water<br>SDG 15: Life on Land<br>SDG 16: Peace and Justice Strong Institutions<br>SDG 17: Partnerships to achieve the Goal					

This question would also provide a link to the website on the SDGs.

15. What would be your main advice for low-income countries (LICs) for achieving UHC? *[open answer format]*

16. Given the coronavirus disease 2019 (COVID-19) pandemic, what would you say are the most critical issues to consider for UHC?
[open answer format]

17. Is UHC (all having access to health care services they need, of sufficient quality to be effective and without facing financial hardship) too ambitious for Uganda?
    a. Yes (free text if yes selected, with open answer text box included)
    b. No
    c. Other (please specify)

18. How urgent do you consider achieving UHC in Uganda to be? *[Likert scale]*
    a. Not urgent, there are other priorities.
    b. Somewhat urgent, to be addressed in next 5-10 years.
    c. Moderately urgent, to be addressed I next 3-5 years.
    d. Very urgent, to be addressed in next 3 year.
    e. Highest priority for health in Uganda, we should implement within 1 year.
    f. N/A

19. Why do you think this is urgent?
    a. To improve health in Uganda.
    b. To reach the SGDs.
    c. To ease to burden on the government of Uganda.
    d. Other (please specify)

20. How many years do you think it will take for Uganda to achieve UHC?
    a. < 1 year.

b. 1-3 years.
c. 3-5 years.
d. 5-10 years.
e. >10 years.
f. Not sure.
g. Other (please specify below)

Final question

21. Would you be willing to participate in an interview, which will be anonymised, as a follow up? If yes, please provide your contact details below.
    [open answer format: this question would be to identify participants for the individual semi-structured interviews.]

Thank you very much for your time and responses.

Strand B: health care worker survey at the local level

2. What is your institution? (*May need to adapt this question for more appropriate version*)
   a. Government
   b. Academia
   c. Private
   d. Other (please specify)

3. Which country is your institution based in?
   a. *Drop down options for countries*.

4. In which region and district is your institution based? (this question is for Ugandans only)
   a. Region (*drop-down options will be provided*)
   b. District (*open answer format*)
   c. Other (please specify)

5. What type of facility do you work in?
   a. Headquarters/government office (non-clinical)
   b. National hospital
   c. Regional referral hospital
   d. HCIV
   e. HCIII, II or I
   f. PNFP or private hospital/clinic

Specific

6. What is most important to you in your day-to-day job? (*potentially multiple selections allowed, and rating will be considered*)
   a. Job satisfaction
   b. Getting paid on time
   c. Career development and progression
   d. Personal development
   e. Safety at work
   f. Serving my community
   g. Management culture
   h. Job security
   i. Other (please specify)

7. Have you heard of universal health coverage (UHC)?
    a. Yes b. No c. Other (please specify)

8. Have you come across UHC in your work?
    a. Yes b. No c. Other (please specify)

9. Which of the following definitions would you say is nearest to UHC?
    a. All people have access to health care services they need.
    b. All people have access to health care services they need, of sufficient quality to be effective.
    c. Some people have access to health care services they need, without facing financial hardship.
    d. All people have access to health care services they need, of sufficient quality to be effective and without facing financial hardship.
    e. All people have free coverage.
    f. Other (please specify)

- *Next page* -

Definition of UHC incorporated onto the page of the next question (9) below:

Universal health coverage (UHC) is defined by the World Health Organization (WHO) as all individuals and communities having access to and being able to use the health services they need, of sufficient quality to be effective, while also ensuring that the use of these services does not expose the individuals to financial hardship.

Source: WHO Fact Sheet (2019) UHC. Available at: https://www.who.int/en/news-room/fact-sheets/detail/universal-health-coverage-(uhc)

10. Do you think that UHC would have an impact on…? *[Likert scale]*

The 17 Sustainable Development Goals (SDGs)	Worsen (1)	No effect (2)	Not sure (3)	Likely/may have an impact (4)	Extremely likely/high impact (5)
SDG 1: No Poverty<br>SDG 2: Zero Hunger<br>SDG 3: Good Health and Well-being<br>SDG 4: Quality Education<br>SDG 5: Gender Equality<br>SDG 6: Clean Water and Sanitation<br>SDG 7: Affordable and Clean Energy<br>SDG 8: Decent Work and Economic Growth<br>SDG 9: Industry, Innovation and Infrastructure<br>SDG 10: Reduced Inequality<br>SDG 11: Sustainable Cities and Communities<br>SDG 12: Responsible Consumption and Production<br>SDG 13: Climate Action<br>SDG 14: Life Below Water<br>SDG 15: Life on Land<br>SDG 16: Peace and Justice Strong Institutions<br>SDG 17: Partnerships to achieve the Goal					

This question would also provide a link to the website on the SDGs.

11. Do you know anyone who works in UHC?
    a. Yes b. No c. Other (please specify)

12. What would you say are some of the barriers to achieving UHC?
    a. Infrastructure
    b. Human resources
    c. Financing
    d. Awareness
    e. Collaboration
    f. Political economy
    g. Information
    h. Other reasons (please specify)

13. Could you provide more detail to your answer for question 9?
    [open answer format]

14. How did you first hear about UHC?
    a. Seminars/Meetings/Conferences
    b. Colleagues
    c. Television
    d. Newspapers
    e. Radio
    f. Internet
    g. Friends/Family
    h. Other (please specify)

15. Are you aware of any strategies from the government or Ministry of Health related to UHC?
    a. Yes b. No c. Other (please specify)

16. Have you heard about any health financing strategy for UHC?
    a. Yes b. No c. Other (please specify)

17. Are you aware of any national targets or goals for UHC?
    a. Yes (please provide information) b. No c. Other (please specify)

18. What level of importance would you value UHC? *[Likert scale]*
    a. Very important
    b. Quite important
    c. Fairly important
    d. Not important
    e. N/A

19. How urgent do you consider achieving UHC in Uganda to be? *[Likert scale]*
    a. Not urgent, there are other priorities.
    b. Somewhat urgent, to be addressed in next 5-10 years.
    c. Moderately urgent, to be addressed I next 3-5 years.
    d. Very urgent, to be addressed in next 3 year.
    e. Highest priority for health in Uganda, we should implement within 1 year.
    f. N/A

20. What is the reasoning for your selection (*optional*)?
    [open answer format]

21. If you do not consider UHC relevant, what other health priority would you say is more important?
    [open answer format]

22. Given the coronavirus disease 2019 (COVID-19) pandemic, what would you say are the most critical issues to consider for UHC?
    [open answer format]

23. Are there any lessons learned from COVID-19 that you could share?
    [open answer format]

24. What is most essential for health systems strengthening in the context of Uganda?
    [open answer format]

Final question

25. Would you be willing to participate in an interview, which will be anonymised, as a follow up? If yes, please provide your contact details with which we can reach you below.
    [open answer format: this question would be to identify participants for the focus group discussions.]

Thank you very much for your time and responses.
