# Supplementary material for: “Sickness has no time”: Awareness and perceptions of health care workers on universal health coverage in Uganda
Source: PLoS One. 2024 Jul 18;19(7):e0306922. doi: 10.1371/journal.pone.0306922 (PMC11257248; doi:10.1371/journal.pone.0306922)
Supplement: S1 Table — (PDF) [file pone.0306922.s002.pdf]

S2 Table. UHC-related responses on knowledge and awareness by health care workers from the online survey (n=274)

Category		Count (as a %)
Knowledge of UHC		
	Yes	181 (66.1)
	No	92 (33.6)
	Other	1 (0.4)
	Total	274 (100.0)
	Details provided for 'other':	
	Via other meetings	1 (0.4)
Selected correct definition of UHC		
	Yes	233 (85.0)
	No	41 (15.0)
	Total	274 (100.0)
How first heard about UHC		
	Internet	160 (36.3)
	Seminars/Meetings/Conferences	90 (20.4)
	Colleagues	50 (11.3)
	Television	39 (8.8)
	Newspapers	33 (7.5)
	Other	33 (7.5)
	Radio	21 (4.8)
	Friends/Family	15 (3.4)
	Total	441 (100.0)
Knowledge of someone who works in UHC		
	Yes	48 (17.5)
	No	223 (81.4)
	Other	3 (1.1)
	Total	274 (100.0)
	Details provided for 'other':	
	Question not clear	2 (0.7)
	At school	1 (0.4)
Awareness of any strategies from government or MoH related to UHC		
	Yes	153 (55.8)
	No	118 (43.1)
	Other	3 (1.1)
	Total	274 (100.0)
Awareness of health financing strategies for UHC		
	Yes	124 (45.3)
	No	146 (53.3)
	Other	3 (1.1)
	Unanswered	1 (0.4)
	Total	274 (100.0)
	Details for 'other' grouping	
	No funding dedicated towards UHC	1 (0.4)

Health insurance	1 (0.4)
Unsure	1 (0.4)
Total	3 (1.1)
Awareness of any national targets or goals for UHC	
Yes	112 (40.9)
No	161 (58.8)
Other	1 (0.4)
Total	274 (100.0)
Details for 'other' grouping	
Not updated	1 (0.4)
