# Supplementary material for: “Sickness has no time”: Awareness and perceptions of health care workers on universal health coverage in Uganda
Source: PLoS One. 2024 Jul 18;19(7):e0306922. doi: 10.1371/journal.pone.0306922 (PMC11257248; doi:10.1371/journal.pone.0306922)
Supplement: S2 Table — (PDF) [file pone.0306922.s003.pdf]

S3 Table. Selected characteristics and UHC-related responses of health care workers by knowledge of UHC (*n*=274)

	Knowledge of UHC (as a percentage, %)				
	Total (*n*=274)	No (*n*=92)	Yes (*n*=181)	Unspecified (*n*=1)	*p*-value
Institution					
Government	154 (56.2)	44 (47.8)	109 (60.2)	1 (100.0)	0.303
Private	11 (4.0)	2 (2.2)	9 (5.0)	0 (0.0)	
Academia	74 (27.0)	31 (33.7)	43 (23.8)	0 (0.0)	
Other	35 (12.8)	15 (16.3)	20 (11.0)	0 (0.0)	
Region (only Ugandan nationals, *n*=270)					
Central	98 (36.3)	31 (34.8)	67 (37.2)	0 (0.0)	0.123
Eastern	26 (9.6)	10 (11.2)	15 (8.3)	1 (100.0)	
Northern	79 (29.3)	26 (29.2)	53 (29.4)	0 (0.0)	
Western	67 (24.8)	22 (24.7)	45 (25.0)	0 (0.0)	
Location					
Rural	80 (29.2)	30 (32.6)	50 (27.6)	0 (0.0)	0.564
Urban	194 (70.8)	62 (67.4)	131 (72.4)	1 (100.0)	
Facility type (for all countries, *n*=274)					
HCIII, II or I	80 (29.2)	30 (32.6)	50 (27.6)	0 (0.0)	0.188
HCIV	45 (16.4)	18 (19.6)	27 (14.9)	0 (0.0)	
Headquarters/Government Office (Non-clinical)	14 (5.1)	1 (1.1)	13 (7.2)	0 (0.0)	
National Hospital	21 (7.7)	10 (10.9)	11 (6.1)	0 (0.0)	
PNFP or Private Hospital/Clinic	39 (14.2)	11 (12.0)	28 (15.5)	0 (0.0)	
Regional Referral Hospital	34 (12.4)	11 (12.0)	22 (12.2)	1 (100.0)	
Other	41 (15.0)	11 (12.0)	30 (16.6)	0 (0.0)	
Selected correct definition of UHC					
Yes	233 (85.0)	70 (76.1)	162 (89.5)	1 (100.0)	0.012
No	41 (15.0)	22 (23.9)	19 (10.5)	0 (0.0)	
Knowledge of someone who works in/for UHC					
Yes	48 (17.5)	1 (1.1)	47 (26.0)	0 (0.0)	<0.001
No	223 (81.4)	91 (98.9)	131 (72.4)	1 (100.0)	

Other	3 (1.1)	0 (0.0)	3 (1.7)	0 (0.0)	
Awareness of any strategies from the government or MoH related to UHC					
Yes	153 (5.8)	28 (30.4)	124 (68.5)	1 (100.0)	<0.001
No	118 (43.1)	69.6	54 (29.8)	0 (0.0)	
Other	3 (1.1)	0 (0.0)	3 (1.7)	0 (0.0)	
Awareness of health financing strategies for UHC					
Yes	124 (45.3)	22 (23.9)	102 (56.4)	0 (0.0)	<0.001
No	146 (53.3)	69 (75.0)	77 (42.5)	0 (0.0)	
Other	3 (1.1)	0 (0.0)	2 (1.1)	1 (100.0)	
Unanswered	1 (0.4)	1 (1.1)	0 (0.0)	0 (0.0)	
Awareness of any national targets or goals for UHC					
Yes	112 (40.9)	21 (22.8)	91 (50.3)	0 (0.0)	<0.001
No	161 (58.8)	71 (77.2)	89 (49.2)	1 (100.0)	
Other	1 (0.4)	0 (0.0)	1 (0.6)	0 (0.0)	
