# Supplementary material for: “Sickness has no time”: Awareness and perceptions of health care workers on universal health coverage in Uganda
Source: PLoS One. 2024 Jul 18;19(7):e0306922. doi: 10.1371/journal.pone.0306922 (PMC11257248; doi:10.1371/journal.pone.0306922)
Supplement: S3 Table — (PDF) [file pone.0306922.s004.pdf]

S4 Table. Selected characteristics and UHC-related responses of health care workers by rural and urban locations of the health facility (*n*=274)

Selected characteristics and UHC-related questions for the health care workers		Number (as a percentage, %)			
		Total (*n*=274)	Rural (*n*=80)	Urban (*n*=194)	*p*-value
Institution					
	Government	154 (56.2)	56 (70.0)	98 (50.5)	0.01
	Private	11 (4.0)	1 (1.3)	10 (5.2)	
	Academia	74 (27.0)	13 (16.3)	61 (31.4)	
	Other	35 (12.8)	10 (12.5)	25 (12.9)	
Facility type					
	HCIII, II or I	80 (29.2)	80 (100.0)	0 (0.0)	<0.001
	HCIV	45 (16.4)	0 (0.0)	45 (23.2)	
	Headquarters/Government Office (Non-clinical)	14 (5.1)	0 (0.0)	14 (7.2)	
	National Hospital	21 (7.7)	0 (0.0)	21 (10.8)	
	PNFP or Private Hospital/Clinic	39 (14.2)	0 (0.0)	39 (20.1)	
	Regional Referral Hospital	34 (12.4)	0 (0.0)	34 (17.5)	
	Other	41 (15.0)	0 (0.0)	41 (21.1)	
Knowledge of UHC					
	Yes	181 (66.1)	50 (62.5)	131 (67.5)	0.564
	No	92 (33.6)	30 (37.5)	62 (32.0)	
	Other	1 (0.4)	0 (0.0)	1 (0.5)	
Knowledge of someone who works in UHC					
	Yes	48 (17.5)	12 (15.0)	36 (18.6)	0.401
	No	223 (81.4)	68 (85.0)	155 (79.9)	
	Other	3 (1.1)	0 (0.0)	3 (1.5)	
Awareness of any strategies from the government or MoH related to UHC					
	Yes	153 (55.8)	44 (55.0)	109 (56.2)	0.974
	No	118 (43.1)	35 (43.8)	83 (42.8)	
	Other	3 (1.1)	1 (1.3)	2 (1.0)	
Awareness of health financing strategies for UHC					
	Yes	124 (45.3)	33 (41.3)	91 (46.9)	0.739
	No	146 (53.3)	45 (56.3)	101 (52.1)	
	Other	3 (1.1)	1 (1.3)	2 (1.0)	
	Missing	1 (0.4)	1 (1.3)	0 (0.0)	
Awareness of any national targets or goals for UHC					
	Yes	112 (40.9)	32 (40.0)	80 (41.2)	0.793
	No	161 (58.8)	48 (60.0)	113 (58.2)	
	Other	1 (0.4)	0 (0.0)	1 (0.5)	
