# Supplementary material for: “Sickness has no time”: Awareness and perceptions of health care workers on universal health coverage in Uganda
Source: PLoS One. 2024 Jul 18;19(7):e0306922. doi: 10.1371/journal.pone.0306922 (PMC11257248; doi:10.1371/journal.pone.0306922)
Supplement: Attachment — Submitted filename: Response.pdf [file pone.0306922.s006.pdf]

Point-by-Point Response to Reviewers

UHC Stakeholders Manuscript | PLOS ONE

Editor comments

Please address the reviewers' comments, including adding further consideration on methods and discussion. You will see that one of the reviewers has suggested improvements on methods and the other one ways of discussing the results.

To ensure the Editor and Reviewers will be able to recommend that your revised manuscript is accepted, please pay careful attention to each of the comments that have been shared with you. Please also consider improving the concluding remarks and policy implication of your work. This way we can avoid future rounds of clarifications and revisions, moving swiftly to a decision.

When submitting your revision, we need you to address these additional requirements.
1. Please ensure that your manuscript meets PLOS ONE's style requirements, including those for file naming. The PLOS ONE style templates can be found at
https://journals.plos.org/plosone/s/file?id=wjVg/PLOSOne_formatting_sample_main_body.pdf and https://journals.plos.org/plosone/s/file?id=ba62/PLOSOne_formatting_sample_title_authors_affiliations.pdf
2. Please remove your figures from within your manuscript file, leaving only the individual TIFF/EPS image files, uploaded separately. These will be automatically included in the reviewers' PDF.

As per one of the reviewers' comments, please ensure that the manuscript has been proofread.

Response and Corrections

Thank you for this opportunity to revise the manuscript and address the reviewers' comments. As advised, each of the comments have been given due attention and addressed within the revised manuscript. All figures have also been removed, the manuscript has been proofread, and corrections have been made.

Finally, all line and page numbers correspond to those in the 'Revised Manuscript with Track Changes' version.

Please find all revisions and accompanying explanations provided below.

Reviewer 1

Overall Comment

This is an interesting article examining the perceptions towards the priorities and importance of UHC among healthcare workers in Uganda. I think this is an important topic for policymakers, researchers and practitioners working on health systems strengthening. However, there are some major shortcomings in several sections of the paper, in particular, how the information is presented as well as discussed. Below are the detailed comments where I point specifically to the limitations, as well as suggestions to improve them.

Response and Corrections

Thank you very much for your insightful and constructive review, and for taking the time to critically examine our manuscript. We very much appreciate your suggestions and have incorporated the specific comments as described in the following sections. Please find below all corrections made.

Specific Comment 1

1) *In the second paragraph of 'study design' sub-section, there is description about the online survey but one key information is lacking: how was the survey designed? Was it based on a prior instrument that has been deployed somewhere else or any published study? Or was it designed from scratch by the researchers? Were the questions validated and adapted to the Ugandan cultural context? I think it would be important to incorporate these information in the study design sub-section as well.*

Response and Correction 1

The survey was designed from scratch and questions were based around the implementation strategy from the WHO Consultative Group on Equity and UHC. This has been clarified in the 'study design' sub-section through the following additions:

- Lines 159-161, page 5: "An original user-friendly, online email survey was developed by the researchers, to examine stakeholders' perceptions of UHC and enhance survey accessibility [15, 17-19]."
- Lines 162-164, page 5: "No relevant prior instrument was found; thus, the online survey was developed by the researchers specifically to answer the specific research questions."

The aim of the survey was to be general and user friendly enough for a wider group of respondents, as the survey was disseminated globally to international stakeholders. However, the interviews and focus group discussion were more culturally specific, and whilst not formally piloted, the FGD was shared with and supported by Ugandan based researchers. This issue was further reflected in the limitations section, as shown below:

- Lines 834-843, page 28: "Furthermore, contrary to the procedure for the online survey, due to time constraints, the qualitative interviews and FGD topic guides were reviewed by Ugandan based team members, not formally piloted. Biases, mistranslations, and a lack of contextual adaptation might have ensued during the ad

hoc translations for the one non-English speaking participant. A pilot test of the guide could have enabled a more contextually and culturally adapted tool. However, piloting was not possible due to the constraints of the timeframe that the pandemic created. English is the national language of Uganda, and all health care worker documentation and trainings are conducted in English. Most qualitative interviews in Kampala with health care workers will be in English. However, sometimes health care workers are more comfortable in their local language and inclusion of a non-English FGD may have been useful."

Specific Comment 2

2) *Table 1 currently appears to be rather messy and overloaded with information. I think the figures can be presented more succinctly and clearly. I think there is no need to spell out categories for 'other' and details for 'other' in the table. Rather, these information can be placed as notes under the table.*

Response and Correction 2

Thank you very much for the helpful idea. This suggestion has been taken on board, categories for 'other' have been moved to the notes section under the table, and Table 1 (lines 340-349, page 12) has now been presented in a more succinct way.

Specific Comment 3

3) *The authors should explain how the radar chart (Figure 3) is interpreted better. I am somewhat confused by the information presented here. How is goal 3 (health and well-being) perceived as very likely to be impacted by UHC by more than half of the respondents? Where does the 57% come from? It is not very intuitive if I look at the radar chart. How about the percentages for Goal 6 and Goal 1? Again, I cannot see these information from the radar chart. Perhaps it is better to present the information in a table format with clear figures instead of a vague radar chart, unless the authors describe clearly how should this radar chart be interpreted by the readers.*

Response and Correction 3

The radar chart has been amended to reflect all percentages and provide the data labels. Please find below the revised Figure 3.

Fig 3. Perceived impact of UHC on the 17 UN SDGs.

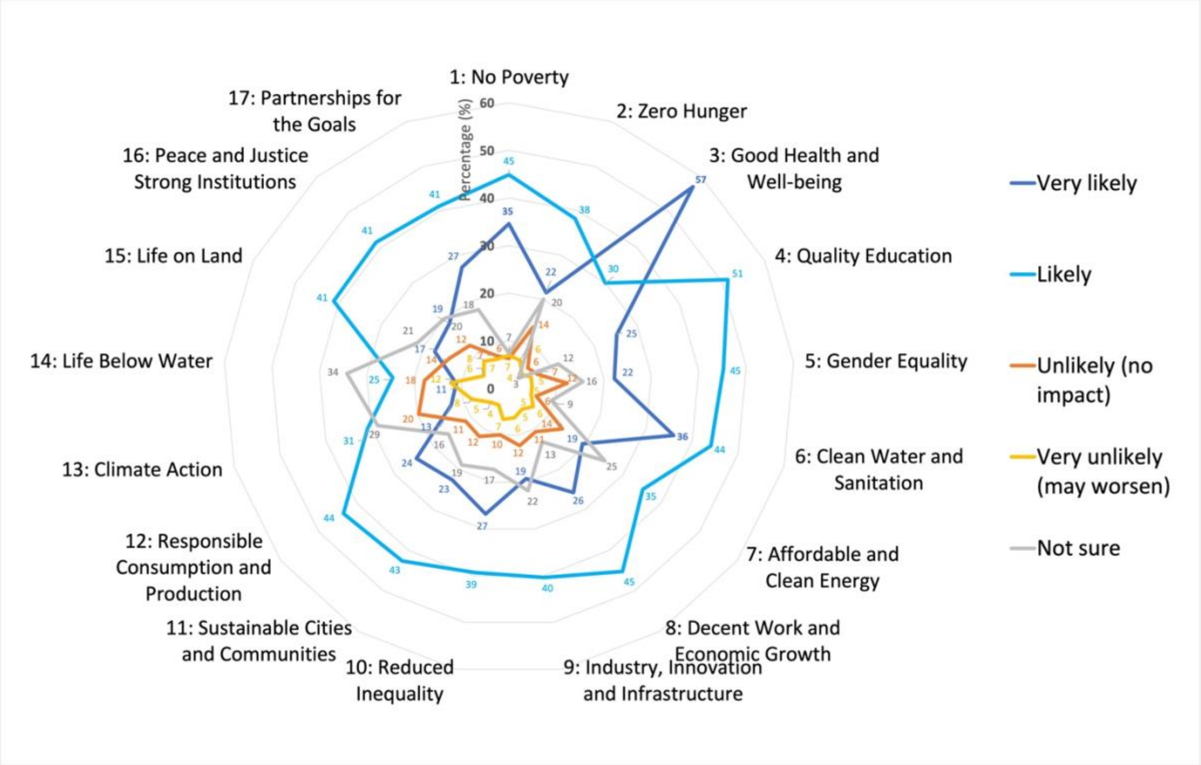

An additional explanation has been included within the figure caption to help with the interpretation of the radar chart:

- Lines 398-403, page 14: "**Fig 3. Perceived impact of UHC on the 17 UN SDGs.** The radar chart displays the outcomes of the 5-point Likert scale, and the datapoints are shown as percentages, whereby the blue lines represent positive responses and the other colours (orange, yellow and grey) symbolise more negative or inconclusive responses. The angular axis (full circle) illustrates each of the SDGs and the radial axis (line of percentages) shows the frequency of responses as a percentage. The peaks show the most prominent choice or highest frequency of responses for each of the SDGs as a percentage."

This figure was included to provide a graphical presentation of the data and to avoid the use of too many tables.

Specific Comment 4
4) *Table 2 seems to appear from nowhere. I am not sure if there is any value presenting the findings in this manner. Perhaps there is no need to present these information. Even if the authors would like to, I would suggest making them as narratives/texts.*
**Response and Correction 4**

Thank you for this suggestion. Table 2 has now been deleted, as recommended. Instead a few of these findings were integrated into the results section, as shown below:

- Lines 407-413, page 15:
  "Insights on the coronavirus disease 2019 (COVID-19) pandemic from open-ended responses:
  Lessons learned from the COVID-19 pandemic included the need for emergency preparedness plans and response readiness appropriate to the country, strengthening health system infrastructure, medical supplies, equipment, and digitalisation, providing sufficient health sector funding for interventions, enhancing HCW remuneration, awareness creation, health promotion and education and the importance of infection prevention and control measures."

Other key findings from the former table were considered redundant and therefore deleted entirely.

Specific Comment 5
5) *I think Table 3 can be presented in a different manner. First, I do not think there the description column is redundant as each of the six building blocks is already quite clear and intuitive to the readers. What can go to the table, instead, would be a summary of the facilitators for and barriers to each of the six building blocks. What the authors can do here is to summarize the narratives in the main text as bullet points here.*
**Response and Correction 5**

Table 3 (now Table 4) has been revised according to the suggestion. The description column was deleted and replaced by two new columns: a summary of the barriers and key messages (facilitators), summarised by the narratives presented in the main text.

Revised Table 4 (*formerly Table 3*) on lines 467-478, pages 17-18, is shown below:

Table 4. Overview of key emerging themes from data on the perceptions and awareness of UHC among stakeholders

Main theme*	Six building blocks of health systems	Barriers (examples derived from data)	Key messages (and facilitators)
**I – Organisation**	Leadership and Governance	• Limited quality health infrastructure<br>• Lack of safe water access<br>• Power outages<br>• Poor physical accessibility of health facilities and referral system<br>• Lack of available services<br>• Procurement issues	1. Health care workers valued through appropriate remuneration<br>2. Efficient cross-sectoral collaboration and cooperation<br>3. Harmonisation of objectives and efforts
	Health Workforce	• Overworked health professionals<br>• Understaffed health facilities<br>• Lack of professionalism impacting health services	
**II – Power**	Financing	• Budgeting issues<br>• Insufficient public financing for health<br>• Downplaying of activities to redivert funds<br>• Mishandling of funds and weak accountability measures	1. Provision of financial resources and accountability of utilisation<br>2. Balance among stakeholders involved<br>3. Awareness of strategies and information power
**III – Communication**	Health Information	• Lack of community sensitisation and involvement in UHC strategies<br>• No apparent communication strategy<br>• Concept of UHC unclear<br>• Lack of grassroots level knowledge of UHC	1. Sensitisation of concept and community inclusion<br>2. Empowerment of own entitlements<br>3. Accessible information on UHC for raising awareness
**IV – Trust**	Access to Medical Products	• Medicine stock outs<br>• Expired and sub-standard medications affecting care<br>• Lack of medical products and equipment	1. Accountability and deliver on promises<br>2. Confidence in receiving intended services<br>3. Receive services needed of good quality
	Service Delivery	• Needed services not received by patient<br>• Lack of quality services and skilled personnel<br>• Access to services costly<br>• Incorrect perception of free health care services	

**Note:* The themes were underpinned by the six health system building blocks conceptualisation [4].

Specific Comment 6
6) In addition to all the findings presented in the article, I find that there might be another interesting analysis which the authors could explore. What are the predictors or factors influencing (i) knowledge of UHC, and (ii) awareness towards UHC? Can regression analyses be performed to tease out what might be some of the demographic, social and institutional factors influencing the above two dependent variables (knowledge and awareness towards UHC)?
**Response and Correction 6**

Thank you for this interesting idea for further analyses. While this would be a fascinating angle to explore, this idea is beyond the scope of the research objective and would have required a different approach, including exploring the literature on predictive analysis and related factors, to ensure validity of findings. In addition, the dataset was considered not extensive enough to conduct a robust regression analysis as suggested. We have added this to the limitations (lines 825-828), in the following way:

- Lines 825-828, page 28: “The low response rate of the survey limits the generalisability. Further exploration of the predictors or factors influencing (i) knowledge of UHC, and (ii) awareness towards UHC through regression analyses would have been interesting, but the data collected was not detailed enough for this to be undertaken.”

Specific Comment 7
7) Discussion section (P23 L470-472), the authors wrote “Financing and political will were considered the most important factors in achieving UHC, and conversely, financing and the political economy were reported as the main barriers.” This sentence is unclear and vague, I do not quite understand what the authors mean here. Please revise.
**Response and Correction 7**

The sentence has been revised to provide more clarity. The following changes have been made:

- Lines 680-682, page 25: “Increased financing and strong political will were considered the most important factors in achieving UHC. Concurrently, limited financing and the political economy were also reported as the main barriers to UHC by the online survey respondents.”

Specific Comment 8
8) *The discussion section is too thin. I think there needs to be deeper reflection on the following points:*<br>a. *implications of findings to the UHC agenda and UHC implementation in Uganda.*<br>b. *What do the findings from this study mean for other low- and middle-income countries (LMIC)? In other words, what can other LMICs learn from your findings?*<br>c. *Generalisability (external validity) of your findings needs to be discussed as well.*<br>d. *What would be some of the policy recommendations in lieu of the findings.*
**Response and Correction 8**

Thank you very much for this helpful comment. The discussion section has been revised and includes more information on the points raised within the comment. Moreover, the limitations section has been separated out and included under a new sub-heading called 'Limitations' (lines 818-862, pages 28-29).

A new section on the public health policy implications and recommendations has been included at the end of the discussion to address comments 'a', 'b', and 'd'. More specifically, the following has been incorporated:

- Lines 864-893, pages 29-31:
  "Public health policy implications and recommendations:
  Most of the key messages delineated from the findings have implications for global health policy and in LMICs. In terms of multisectoral collaboration practices and a systems thinking approach required for UHC, power and trust were considered key constituents. To safeguard trust, robust accountability mechanisms, a guarantee of fulfilment of promises or objectives and, most importantly, transparency and honesty are important considerations for the delivery of any service or strategy. In the same regard, accessible and continuous communication at various levels, considering power dynamics between local communities and national entities, remains pivotal to achieve transparency, sensitisation, and a greater awareness of UHC principles.

  Participants in this study identified the absence of a comprehensive and well-defined communication strategy for UHC to enable more widespread awareness. Such a communication strategy could benefit from the support of an established technical working group in implementing the already defined UHC Roadmap. Equally, one or more focal points for UHC in order to help guide developments, provide a main access point for the different levels of stakeholders, and actively promote the dialogue among these key stakeholders could help move the strategies forward. The focal point would also be instrumental in implementing the communication strategy, in quality control and disseminating related public health messaging for UHC.

  Finally, despite the multitude of different stakeholders involved in UHC and the enormity of this collaboration, it remains imperative to consider and value those individuals providing the muscle of health service delivery, the HCWs. It is important

to be mindful of personal motivational factors of individuals and incorporating these into strategies for improved ownership, empowerment, and implementation. Protecting and nurturing the 'heart' or reason behind the work HCWs perform could help improve relationships and satisfaction in the working environment. To develop adequate, competent, and motivated personnel who are equitably distributed across health facilities in the country, appropriate remuneration and sufficient financing need to be accounted for."

To address comment 'c' the following has been added within the limitations section:

- Lines 823-825, page 28: "Additionally, issues of representativeness and generalisability to other low-income countries need to be considered. The low response rate of the survey limits the generalisability."

Finally, to expand the discussion and provide a deeper reflection on the suggested comments, the text below has been included:

- Lines 764-768, page 26: "Evidence from other countries in the region, such as Botswana, Eswatini, the Seychelles, or South Africa, within the literature have highlighted the benefits of high political commitment and accountability, increasing domestic financing for UHC, investing in PHC and incremental changes for the advancement of population health [33-38]."

- Lines 772-776, page 26: "Topp and colleagues argued that more research is needed on power in the field of health policy and systems and this empirical research aimed to provide further insight into this knowledge gap [39]. Ssennyonjo [40] highlighted the concept of ideational power, its influence in form of groupthink in political environments and the bidirectional influence of structure and agency in health financing reforms in Uganda."

- Lines 181-182, page 26: "a disconnect between top-level district officials and lower-level staff exacerbating policy-to-implementation gaps, echoed by the literature [14,41]."

- Lines 789-801, page 27: "In view of the projected global shortage of 18 million HCWs by 2030 [43], findings from this analysis touched on neglected frontline health workers confronted with overburdened and understaffed health facilities, referral system inefficiencies and a lack of proper remuneration. Participants agreed that confidence in receiving intended services of quality was central to the development of trust. This finding was corroborated in the literature, whereby a systematic review of stockouts, a complete absence or unavailability of medical products, including medicines and vaccines, among community health workers in low- and middle-income countries (LMICs) suggested an increase in stockout rates from 2016 to 2021, noting that procurement and distribution issues, such as governance and coordination problems, were linked with trust issues among the population [44, 45]. Although subtleties related to the level of trust in health settings is difficult to measure, earlier research on the conceptualisation of trust has suggested a relationship between trust and knowledge, in the sense that trust may promote timely information sharing [46, 47]."

- Lines 804-806, page 27: “Particularly, the perception that UHC was a vague concept, which was not easily understood, and failed to reach the grassroots level was a concern shared by many participants.”

- Lines 808-811, page 27: “This is reinforced within the literature, whereby a cross-sectional online survey of 326 nurses in Hong Kong conducted by Tung et al. 2016 revealed that nurses being relatively indifferent to health policies created knowledge gaps that affected the success of UHC initiatives [15].”

Specific Comment 9
9) *This article needs to be edited more carefully. I spotted some grammatical mistakes throughout the paper, just to highlight a few more obvious one:-*<br>a. *P.4 L97 ‘focussed’ should be ‘focused’.*<br>b. *P.5L140 ‘The final online survey was comprised of an introductory section…’ should be ‘The final online survey comprised an introductory section…”*<br>c. *P.9 L239-240, the authors wrote ‘Most respondents were based in an urban setting, as opposed to a rural one, which was derived from the health facility location’. This sentence is very convoluted, can be simplified as ‘most respondents were based in an urban setting’. What do you mean by ‘derived from the health facility location’? Please clarify and revise.*<br>d. *P.24 L499: Shouldn’t ‘considering these limitations’ be ‘despite these limitations’?*
**Response and Correction 9**

Thank you for these edits. All suggested changes have been incorporated and the paper has been revised to address any further grammatical mistakes throughout the paper.

The following amendments have been made:

- Line 115, page 4: ‘focused’
- Line 134, page 4: ‘… on HCW perceptions of UHC’
- Line 135, page 4: ‘… affect their attitudes towards…’
- Line 155, page 5: ‘… conducted between 1 and 5 February 2021’
- Line 178, page 6: ‘The final online survey comprised an introductory section…”
- Line 180, page 6: ‘closed-ended’
- Line 221, page 7: ‘… further participation in form of an interview.’
- Line 240, page 8: ‘… conducted between mid-February to the end of April 2022.’
- Lines 330-331, page 11: ‘Most respondents were based in an urban setting. Both urban and rural settings were derived from the health facility location.’
- Line 387, page 14: ‘all HCWs valued…’
- Line 412, page 15: ‘remuneration’
- Line 502, page 22: ‘A negative sentiment towards the clarity of UHC arose…’
- Line 632, page 23: ‘… expressed he was prescribed forced…’
- Line 669, page 26: ‘Despite these limitations…’
- Line 813, page 27: ‘… different localities is needed’

Reviewer 2

Overall Comment

The topic is interesting because UHC is a concept that need to be understood by the implementers. The way the implementers will understand UHC will affect its implementation.
However there are several clarifications that authors need to provide mainly in the method and discussion sections.

Response and Corrections

Many thanks for the helpful feedback and recommendations and comments, which have helped refine the current manuscript. Hopefully, the comments provided have been correctly addressed in the changes made to the manuscript. Please find these changes described below.

Specific Comment 2.1

Title
the title needs to be reformulate to reflect the content. I suggest a title : Awareness and perceptions of health care workers on universal health coverage in Uganda

Response and Correction 2.1

The quote in the title ("Sickness has no time") is meant as a metaphor to signify the risk of becoming sick at any point in time. This quote was taken directly from a study participant and was considered poignant, as it can be applied to the entire concept of UHC and the need for strengthened health systems globally. Anyone can be struck down by a sudden illness at any given time. The added benefit of this quote is that it could be considered captivating and enhance readership. Finally, the quote is highlighted again within the conclusion and was selected to complete the argument made within the discussion section.

The remaining part of the title is the same as the title suggested by the reviewer, and therefore, hopefully, would avoid any potential confusion:

- “Sickness has no time”: Awareness and perceptions of health care workers on universal health coverage in Uganda

Specific Comment 2.2

Introduction

From Line 102 to 106. The authors presented the results of a study in Asia. I suggest to present results from study in Africa related to UHC perceptions such as Koon AD, et al 2017.

Response and Correction 2.2

Thank you for this suggestion. The study in Asia was referred to, rather than the Koon et al. study, because it similarly conducted an online survey. However, as suggested, the results of the study in Asia have now been deleted and replaced by those of the study in Africa by Koon et al. and the following information below has been added to the introduction:

- Lines 120-124, page 4: "In 2016, Koon et al. [14] conducted semi-structured interviews with 60 nurses based in three health facilities in Kenya. Findings revealed that nurses had not heard of UHC and were unfamiliar with the concept. The authors argued that nurses should be involved as stakeholders for successful adoption of UHC initiatives."

Specific Comment 2.3

Method

Study design

The use of mix method is not clear. What is the sequence of utlization and how each of these studies are linked ?

Response and Correction 2.3

The mixed methods design included an online survey to begin with, followed by semi-structured interviews and a focus group discussion (FGD). The online survey was linked to the interview and discussion in that it helped identify individuals who participated in the interviews and FGD. These details were described within the methods section. Hopefully, through the additional edits, these details have been clarified.

Specific Comment 2.4

The online survey was sent to the entire sub-Saharan African region (line 151) but the scope of the study was uganda, why did you select these sample from sub saharan african region. How did the author estimate the sample size for the online survey ?

Response and Correction 2.4

The scope of the online survey was larger than that of the individual interviews and the focus group discussion, as it aimed to capture insights from global and regional experts related to UHC in Uganda. The purpose of this paper was to share the results of the perceptions and awareness of health care workers on UHC in Uganda, because this was the focus of the larger project. This paper is part of a greater PhD project, which examined the Sub-Saharan African region and then focused in on Uganda. The findings related to the full study scope will soon be published in the final thesis.

Specific Comment 2.5
How did the author estimate the sample size for the online survey ?
**Response and Correction 2.5**

The details of the sample size estimation were not included within this manuscript, as it refers to the full research project and is provided within the thesis.

The following information has been added to the methods section:

- Lines 185-202, pages 6-7: "For the online email survey to all stakeholders, which was based on the entire research project, the following sample size calculation was incorporated. The calculation was based on the primary objective of the importance of UHC, which was measured by a 5-point Likert scale question [21, 22]. Given the estimated population size of 1,000, which is an approximation and best estimate based on the distribution list and purposive sampling, the Yamane's sample size determination formula (1967) was applied and adapted by the consideration of a response rate of 95% [23].

$$\text{Sample size } (n) = \frac{N}{1 + N(e^2)} \times \frac{1}{r}$$

$$n = \frac{1000}{1 + 1000(0.05^2)} \times \frac{1}{0.95}$$

$$n = \frac{1000}{3.5} \times 1.05$$

$$n = 300.7518 \ldots$$

$$n = 301$$

$n =$	Desired sample size
$N =$	Total population of participants in distribution list/UHC work
$e =$	precision (0.05)
$r =$	response rate at 95%

In this abovementioned formula, where a 95% confidence level and *p*=0.5 were assumed, '*n*' is the sample size, '*N*' is the population size and '*e*' is the level of precision at 0.05 [24]. Therefore, applying this formula to the parameters of the project produced a sample size of *n*=301 participants. This sample size aimed to address questions related to the perceived importance of UHC, for which response options included "very important", "quite important", "fairly important", "not important", or "not applicable"."

References included:

21. Horst SJ, Pyburn EM. The SAGE Encyclopedia of Educational Research, Measurement, and Evaluation. 2018 2023/01/06. Thousand Oaks, California: SAGE Publications, Inc. Available from: https://methods.sagepub.com/reference/the-sage-encyclopedia-of-educational-research-measurement-and-evaluation.
22. Likert R. A technique for the measurement of attitudes. Archives of Psychology. 1932.
23. Yamane T. Statistics: An Introductory Analysis, 2nd Ed, Harper and Row, New York. 1967.
24. Israel GD. Determining Sample Size. 2013.

Specific Comment 2.6
They sent too many email to many individual, how did they insure that there was not double entry.
**Response and Correction 2.6**

The individual emails were captured and tracked through the use of an external Excel list of contacts, which listed all email addresses and guaranteed that there were no duplications. For the online survey, anonymised responses were guaranteed but information related to the IP addresses made it possible to check for any duplications. Following this process, information related to the IP address was then deleted to safeguard anonymity.

Overall, *n*=6 responses were excluded as three did not provide informed consent and the other three were duplications, where participants completed the survey twice. In those circumstances, the first response was selected and duplications were deleted to avoid desirability bias.

For added clarity, the following sentence has been added to the methods section:

- Lines 211-213, page 7: "Individual emails were captured and tracked through the use of an external Excel list of contacts, which listed all email addresses and guaranteed that there were no duplications."

Specific Comment 2.7
About the sampling of the in-depth semi-structured key informant interviews, from line 164 to 165 authors stated that « the in-depth semi-structured key informant interviews were selected through purposive ». That is in contracdiction with what they stated on line 173 « Further interviewees were identified through the snowballing technique to attempt to expand the breadth of informants included. » Please explain how the interviewees were selected.
**Response and Correction 2.7**

Details on how the interviewees were selected was included through the additional paragraph below:

- Lines 225-233, page 8: "Purposive sampling was used to begin with, during the online survey stage, and snowball sampling was conducted subsequently throughout the data collection phase and through discussions with experts, research team members and participants who were able to suggest further experts who were relevant participants considering our research questions. Additional interviewees were identified through the snowballing technique to expand the breadth of informants included. Both methods were used to expand the reach of relevant participants and to save time, which were considered as strengths of using both approaches together. However, weaknesses exist with this pragmatic approach, which are discussed in the limitations section."

Specific Comment 2.8

How did the authors check the saturation ? Please explain.

Response and Correction 2.8

More information on how saturation was defined and obtained has been added with the addition below:

- Lines 255-258, page 9: "The notion of saturation, defined by Glaser and Strauss (1967) in their seminal work on grounded theory, rested on the concept that new observations added to the main analysis and once these became repetitive (i.e. no new themes emerging), saturation was reached [27]."

Specific Comment 2.9

Why did you use semi structured key informant interviews and focus group discussion. What was the add value of each technique ? Especially because the questions for the FGD were drew from the online survey like the individual semi structured interviews. In adition there was only 1 FGD. Please explain why did you conduct only one FGD ?

Response and Correction 2.9

Three different data capture methods (the online survey, semi-structured in-depth interviews, and the FGD) were used because of the strengths and weaknesses of each.

First, the online survey was chosen in order to maximise the reach and breadth of multisectoral stakeholders. This method intended to provide access to global, regional, and local experts from a multitude of sectors. The anonymised approach also enabled the possibility to ask more sensitive questions (for example, whether UHC was too ambitious for Uganda). Despite strengths, such as being a short survey, that was user-friendly and easy to complete, and a cost-effective approach, there were inevitable weaknesses. These included potential for low response rate, online survey fatigue, lack of time, issues with internet access and a brief survey to the detriment of further detail. More weaknesses are discussed in detail in the limitations section.

Second, the semi-structured in-depth interviews aimed to address the issues of the online survey, by providing more focused and detailed perspectives. Topics could be explored in greater detail and one-on-one conversations enabled more personalised responses to better understand nuances. On the other hand, weaknesses of this approach included response and subjective bias, and issues related to lack of availability of respondents.

Third, the focus group discussion was selected to observe a group discussion for further nuances in perspectives and personalised opinions. However, weaknesses such as the impact of group dynamics and more dominant individuals speaking more often and creating a lack of balance of opinions were evident.

Only one FGD was conducted given the time constraints related to the fieldwork and the fact that only one FGD was planned for within the project proposal. More effective planning and additional FGDs could have been useful to obtain more relevant and reliable findings.

Specific Comment 2.10

The population study was not clearly defined for each survey . Also the selection was not clear

Response and Correction 2.10

The study population was clarified in the methods section, as shown below:

- Lines 153-156, page 5: "The study population of the online survey included local, national, regional, and international stakeholders who worked in UHC, health policy, financing, and systems research. For the semi-structured interviews and FGD, the study population included Ugandan health care workers."

The population included experts from international organisations, regional and national organisations, and institutes. More details on the selection of participants were described in the following paragraphs in the methods section.

Specific Comment 2.11

Results

-How many individual interviews were conducted ? how many parcipants did you have in FGD ? What were their background ? Even if youd id not collect their name , information related to their professionnal category, organization, level of facility could be collected.

Response and Correction 2.11

More information on the number of interviews conducted, number of participants in the FGD and background details have been included in the results section, both descriptively and in form of two additional tables. The following additions have been made:

- Lines 416-451, page 15:
  "**Semi-structured in-depth interviews and focus group discussion (FGD)**
  Overall, 23 HCWs took part in the semi-structured interviews (Table 2) and eight HCWs participated in the focus group discussion (Table 3). Out of the total participants, 10 (32%) of participants were female and most (84%) worked in government institutions. Further demographic information beyond that listed in Tables 2 and 3 was not collected to safeguard the anonymity of respondents."

Table 2. Selected socio-demographic data of health care workers comprising the semi-structured interview group (*n*=23)

Count	Level	Institution	Type of organisation or facility	Sex
1	National	Government	Research Institute	Male
2	National	Government	National Referral Hospital	Male
3	National	Government	Health Centre III	Female
4	National	Government	Private Hospital	Male
5	National	Government	Laboratory	Male
6	National	Government	Health Centre (undisclosed number)	Female
7	National	Government	Laboratory	Male
8	National	Government	Laboratory	Male
9	National	Government	Research Institute	Male
10	National	Government	National Referral Hospital	Male
11	National	Government	General Hospital	Male
12	National	Government	Laboratory	Male
13	National	Government	Health Centre IV	Male
14	National	Government	National Referral Hospital	Male
15	National	Government	Public Health Institute	Male
16	National	Government	Research Institute	Male
17	National	Government	General Hospital	Male
18	National	Government	Research Institute	Male
19	National	Non-Governmental Organisation	Humanitarian Organisation	Male
20	National	Non-Governmental Organisation	Clinic	Male
21	National	Non-Governmental Organisation	International Organisation (Field Office)	Male
22	National	Non-Governmental Organisation	Network	Male
23	National	Private	Clinic	Male

Table 3. Selected socio-demographic data of health care workers in the focus group discussion (*n*=8)

Count	Level	Institution	Type of facility	Role	Profession	Sex
1	National	Government	Health Centre	Nursing Officer	Health care worker	Female
2	National	Government	Health Centre	Counsellor	Health care worker	Female
3	National	Government	Health Centre	Midwife	Health care worker	Female
4	National	Government	Health Centre	Midwife	Health care worker	Female
5	National	Government	Health Centre	Linkage Facilitator	Health care worker	Female
6	National	Government	Health Centre	Linkage Facilitator	Health care worker	Female
7	National	Government	Health Centre	Clinical Officer	Health care worker	Female
8	National	Government	Health Centre	Peer mother/ Counsellor	Health care worker	Female

Specific Comment 2.12
-On line 323, the authors stated that « None of the FGD participants had heard of UHC prior to the discussion, ». That is surprising because the praticipants for the FGD were drawn from the online survey. How could you explain this situation ?
**Response and Correction 2.12**

The sentence has been clarified through the following change:

- Lines 480-482, page 18: "None of the FGD participants had heard of UHC prior to the discussion and participating in the survey, thus the guiding probes specific to UHC were re-formulated into questions related to health service access and provision, and financial implications in Uganda."

This situation could be explained through issues related to the FGD methodology. These issues have been incorporated within the limitations section of the discussion, as below:

- Lines 834-843, pages 28-29: "Furthermore, contrary to the procedure for the online survey, due to time constraints, the qualitative interviews and FGD topic guides were reviewed by Ugandan based team members, not formally piloted. Biases, mistranslations, and a lack of contextual adaptation might have ensued during the ad hoc translations for the one non-English speaking participant. A pilot test of the guide could have enabled a more contextually and culturally adapted tool. However, piloting was not possible due to the constraints of the timeframe that the pandemic created. English is the national language of Uganda, and all health care worker documentation and trainings are conducted in English. Most qualitative interviews in Kampala with health care workers will be in English. However, sometimes health care workers are more comfortable in their local language and inclusion of a non-English FGD may have been useful."

Specific Comment 2.13
The categorization of the six building blocks into the main themes is not clear because some themes such as trust or communication are backbone of challenges in the health system. Why did you choose to categorise like this ? I suggest to revise the presentation by looking at the main theme for each block.
**Response and Correction 2.13**

The categorisation was selected, because the six building blocks framework is widely known in health systems research. While the themes are interlinked and do provide the backbone of challenges in the health system, the key messages selected were considered to feature more prominently within the cross-cutting building blocks. The themes have been expanded more in the revised Table 4 (formerly Table 3), which hopefully addresses further concerns.

Specific Comment 2.14
Discussion   *The discussion is confusing and not reflect the results. I suggest a deep revision of this section taking into account the results and interpretations. To reinforce the discussion, a literature review will be helpful on the different themes and results.*
**Response and Correction 2.14**

The discussion section has now been revised to reflect the results more closely. More study implications have been discussed, discussion of the relevant literature was added to provide additional insights into the different themes and results, and additions were made, which are reflected in '<u>Response and Correction 8</u>' detailed above.

Further discussion of relevant literature was included through the following additions made:

- Lines 181-182, page 26: "a disconnect between top-level district officials and lower-level staff exacerbating policy-to-implementation gaps, echoed by the literature [14,41]."

- Lines 764-768, page 26: "Evidence from other countries in the region, such as Botswana, Eswatini, the Seychelles, or South Africa, within the literature have highlighted the benefits of high political commitment and accountability, increasing domestic financing for UHC, investing in PHC and incremental changes for the advancement of population health [33-38]."

- Lines 772-776, page 26: "Topp and colleagues argued that more research is needed on power in the field of health policy and systems and this empirical research aimed to provide further insight into this knowledge gap [39]. Ssennyonjo [40] highlighted the concept of ideational power, its influence in form of groupthink in political environments and the bidirectional influence of structure and agency in health financing reforms in Uganda."

- Lines 789-801, page 27: "In view of the projected global shortage of 18 million HCWs by 2030 [43], findings from this analysis touched on neglected frontline health workers confronted with overburdened and understaffed health facilities, referral system inefficiencies and a lack of proper remuneration. Participants agreed that confidence in receiving intended services of quality was central to the development of trust. This finding was corroborated in the literature, whereby a systematic review of stockouts, a complete absence or unavailability of medical products, including medicines and vaccines, among community health workers in low- and middle-income countries (LMICs) suggested an increase in stockout rates from 2016 to 2021, noting that procurement and distribution issues, such as governance and coordination problems, were linked with trust issues among the population [44, 45]. Although subtleties related to the level of trust in health settings is difficult to measure, earlier research on the conceptualisation of trust has suggested a relationship between trust and knowledge, in the sense that trust may promote timely information sharing [46, 47]."

Additional references:

33. Gatome-Munyua A, Sieleunou I, Sory O, Cashin C. Why Is Strategic Purchasing Critical for Universal Health Coverage in Sub-Saharan Africa? Health Systems & Reform. 2022;8(2):e2051795. doi: https://doi.org/10.1080/23288604.2022.2051795.
34. Jaca A, Malinga T, Iwu-Jaja CJ, Nnaji CA, Okeibunor JC, Kamuya D, et al. Strengthening the Health System as a Strategy to Achieving a Universal Health Coverage in Underprivileged Communities in Africa: A Scoping Review. International Journal of Environmental Research and Public Health. 2022;19(1):587. doi: https://doi.org/10.3390/ijerph19010587. PubMed PMID: 35010844.
35. Piatti-Fünfkirchen M, Lindelow M, Yoo K. What Are Governments Spending on Health in East and Southern Africa? Health Systems & Reform. 2018;4(4):284-99. doi: https://doi.org/10.1080/23288604.2018.1510287.
36. Workie NW, Shroff E, Yazbeck AS, Nguyen SN, Karamagi H. Who needs big health sector reforms anyway? Seychelles' road to UHC provides lessons for Sub-Saharan Africa and Island nations. Health Systems and Reform. 2018;4:362-71. doi: https://10.1080/23288604.2018.1513265.
37. Ataguba JE, McIntyre D. The incidence of health financing in South Africa: findings from a recent data set. Health Economics, Policy and Law. 2017;13(1):1-24. doi: https://dx.doi.org/10.1017/S1744133117000196.
38. Pagiwa V, Shiell A, Barraclough S, Seitio-Kgokgwe O. A Review of the User Fees Policy for Primary Healthcare Consultations in Botswana: Problems With Effective Planning, Implementation and Evaluation. International Journal of Health Policy and Management. 2022;11(10):2228-35. doi: https://doi.org/10.34172/ijhpm.2021.141.
39. Topp SM, Schaaf M, Sriram V, Scott K, Dalglish SL, Nelson EM, et al. Power analysis in health policy and systems research: a guide to research conceptualisation. BMJ Global Health. 2021;6(11):e007268. doi: https://doi.org/10.1136/bmjgh-2021-007268.
40. Ssennyonjo A. Beyond "Lack of Political Will": Elaborating Political Economy Concepts to Advance "Thinking and Working Politically"; Comment on "Health Coverage and Financial Protection in Uganda: A Political Economy Perspective". International Journal of Health Policy and Management. 2022. doi: https://doi.org/10.34172/ijhpm.2022.7297.
41. Okech TC, Lelegwe SL. Analysis of Universal Health Coverage and Equity on Health Care in Kenya. Global Journal of Health Science. 2015;8(7):218-27. doi: https://dx.doi.org/10.5539/gjhs.v8n7p218.
43. Boniol M, Kunjumen T, Nair TS, Siyam A, Campbell J, Diallo K. The global health workforce stock and distribution in 2020 and 2030: a threat to equity and 'universal' health coverage? BMJ Global Health. 2022;7(6):e009316. doi: https://doi.org/10.1136/bmjgh-2022-009316.
44. Olaniran A, Briggs J, Pradhan A, Bogue E, Schreiber B, Dini HS, et al. Stock-outs of essential medicines among community health workers (CHWs) in low- and middle-income countries (LMICs): a systematic literature review of the extent, reasons, and consequences. Human Resources for Health. 2022;20(1):58. doi: https://doi.org/10.1186/s12960-022-00755-8.
45. World Health Organization. Meeting Report: Technical Definitions of Shortages and Stockouts of Medicines and Vaccines. 2017.
46. Connell NA, Mannion R. Conceptualisations of trust in the organisational literature: some indicators from a complementary perspective. Journal of Health Organization and Management. 2006;20(5):417-33. Epub 2006/11/08. doi: https://doi.org/10.1108/14777260610701795. PubMed PMID: 17087403.
47. Kramer RM, Cook KS. Trust and distrust in organizations: Dilemmas and approaches. New York, NY, US: Russell Sage Foundation; 2004. xii, 381-xii
