# Supplementary material for: “Sickness has no time”: Awareness and perceptions of health care workers on universal health coverage in Uganda
Source: PLoS One. 2024 Jul 18;19(7):e0306922. doi: 10.1371/journal.pone.0306922 (PMC11257248; doi:10.1371/journal.pone.0306922)
Supplement: Attachment — Submitted filename: review PLOs One.docx [file pone.0306922.s005.docx]

**General comments**

The topic is interesting because UHC is a concept that need to be understood by the implementers. The way the implementers will understand UHC will affect its implementation.

However there are several clarifications that authors need to provide mainly in the method and discussion sections.

**Title**

the title needs to be reformulate to reflect the content. I suggest a title : Awareness and perceptions of health care workers on universal health coverage in Uganda

**Introduction**

From Line 102 to 106. The authors presented the results of a study in Asia. I suggest to present results from study in Africa related to UHC perceptions such as Koon AD, et al 2017.

**Method**

Study design

The use of mix method is not clear. What is the sequence of utlization and how each of these studies are linked ?

The online survey was sent to the entire sub-Saharan African region (line 151) but the scope of the study was uganda, why did you select these sample from sub saharan african region. How did the author estimate the sample size for the online survey ?

They sent too many email to many individual, how did they insure that there was not double entry.

About the sampling of the in-depth semi-structured key informant interviews, from line 164 to 165 authors stated that « the in-depth semi-structured key informant interviews were selected through purposive ». That is in contracdiction with what they stated on line 173 « Further interviewees were identified through the snowballing technique to attempt to expand the breadth of informants included. » Please explain how the interviewees were selected.

How did the authors check the saturation ? Please explain.

Why did you use semi structured key informant interviews and focus group discussion. What was the add value of each technique ? Especially because the questions for the FGD were drew from the online survey like the individual semi structured interviews. In adition there was only 1 FGD. Please explain why did you conduct only one FGD ?

The population study was not clearly defined for each survey . Also the selection was not clear.

**Results**

-How many individual interviews were conducted ? how many parcipants did you have in FGD ? What were their background ? Even if youd id not collect their name , information related to their professionnal category, organization, level of facility could be collected.

-On line 323, the authors stated that « None of the FGD participants had heard of UHC prior to the discussion, ». That is surprising because the praticipants for the FGD were drawn from the online survey. How could you explain this situation ?

The categorization of the six building blocks into the main themes is not clear because some themes such as trust or communication are backbone of challenges in the health system. Why did you choose to categorise like this ? I suggest to revise the presentation by looking at the main theme for each block.

Discussion

The discussion is confusing and not reflect the results. I suggest a deep revision of this section taking into account the results and interpretations. To reinforce the discussion, a literature review will be helpful on the different themes and results.
